# Phone-based screening versus home-based screening after tuberculosis in India (TB Aftermath): a multicentre, open-label, randomised, controlled, non-inferiority trial

**DOI:** 10.1016/j.langlo.2026.103971

**Published:** 2026-07-02

**Authors:** Samyra R Cox, Abhay Kadam, Shahanara Valawalkar, Gayatri Lele, Aye Hnin Moe, Vipul Dhamale, Akshay N Gupte, Nishi Suryavanshi, Emily A Kendall, Hojoon Sohn, Christopher J Hoffmann, Arjunlal Kakrani, Ryan Thompson, Nikhil Gupte, Madhu Sudan Barthwal, Jonathan E Golub, Vidya Mave

**Affiliations:** Department of International Health, Johns Hopkins Bloomberg School of Public Health, Baltimore, MD, USA (S R Cox PhD MPH, Prof J E Golub PhD MPH); Johns Hopkins India, Center for Infectious Diseases in India (CIDI), Pune, India (A Kadam MSc, S Valawalkar MBBS MPH, G Lele MSc, N Suryavanshi PhD, N Gupte PhD, V Mave MD MPH); Division of Infectious Diseases, Johns Hopkins School of Medicine, Baltimore, MD, USA (A H Moe MBBS, MPH, N Suryavanshi, E A Kendall MD PhD, C J Hoffmann MD MPH, N Gupte, Prof J E Golub, V Mave); Department of Respiratory Medicine, Dr D Y Patil Medical College, Hospital and Research Centre, Pune, India (V Dhamale MSc, Prof M S Barthwal MD); Boston University School of Public Health, Global Health, Boston, MA, USA (A N Gupte PhD MBBS); Department of Preventive Medicine, Seoul National University College of Medicine, Seoul, South Korea (H Sohn PhD MPH); Department of Medicine, Dr D Y Patil Medical College, Hospital and Research Centre, Pune, India (Prof A Kakrani MD); Department of Global Health and Population, Harvard TH Chan School of Public Health, Boston, MA, USA (R Thompson MSPH)

## Abstract

**Background:**

Tuberculosis survivors and their household contacts are at increased risk for tuberculosis even after treatment completion; however, effective strategies for post-tuberculosis screening remain untested. We aimed to assess whether phone-based screening is non-inferior to home-based screening for tuberculosis after treatment completion in India.

**Methods:**

In this multicentre, open-label, randomised, controlled, non-inferiority trial, we enrolled tuberculosis survivors (aged ≥18 years) within 60 days of completing treatment for any form of tuberculosis from six public clinics in Maharashtra, India. Household contacts were enumerated at baseline (all ages). Survivors were randomly assigned (1:1) to receive phone-based or home-based symptom screening at 6 months and 12 months. The trial was powered to identify a difference in the tuberculosis detection rate per 100 person-years among survivors and contacts together (primary outcome), comparing screening groups during the 12 months after survivor treatment completion. We conducted an intention-to-treat analysis with a rate difference non-inferiority margin of 1·70 per 100 person-years based on the upper bound of the 95% CI. We adjusted for household-level clustering of tuberculosis by fitting a multilevel Poisson model. We also conducted a subgroup analysis of detection rates among survivors and contacts separately. This study is registered with ClinicalTrials.gov, NCT04333485 and is completed.

**Findings:**

We enrolled 1076 tuberculosis survivors (537 in the phone-based screening group and 539 in the home-based screening group) and enumerated 3309 contacts (1638 in the phone-based screening group and 1671 in the home-based screening group) between Jan 21, 2021, and Sept 4, 2023. In the 12-month post-treatment period, tuberculosis was detected in 75 (7%) tuberculosis survivors and 30 (1%) contacts: 41 events in the phone-based screening group (rate 1·19 [95% CI 0·82–1·71]) compared with 64 events in the home-based screening group (1·92 [1·37–2·45]). Non-inferiority was reached (rate difference 0·73 [95% CI 0·05–1·41]). Among tuberculosis survivors, the recurrence detection rate was 4·36 (95% CI 2·87–6·34) in the phone-based screening group compared with 8·00 (5·90–10·60) in the home-based screening group (p=0·01). Among contacts, the tuberculosis detection rate was 0·61 (95% CI 0·32–1·07) in the phone-based screening group compared with 0·70 (0·39–1·20) in the home-based screening group (p=0·70).

**Interpretation:**

Risk of tuberculosis remains high for households with a tuberculosis survivor. For detecting tuberculosis among survivors and their household contacts together, phone-based screening was non-inferior to home-based screening. For survivors, the recurrence detection rate was higher in the home-based screening group. Countries with a high burden of recurrence should consider home-based screening among tuberculosis survivors.

## Introduction

In 2024, an estimated 10·7 million people developed tuberculosis and 1·23 million people died from the disease.^1^ To find and treat tuberculosis earlier, WHO recommends systematically screening household contacts of people with tuberculosis disease. This screening intervention is usually implemented shortly after the index patient is diagnosed.^2^ However, contacts remain at increased risk for tuberculosis even after the index patient completes treatment.^3^ Additionally, the household member who survived tuberculosis is at risk for both endogenous reactivation (relapse) and exogenous reinfection.^4^ Both forms of recurrent tuberculosis are associated with poor outcomes.^5,6^ Household contacts have the highest risk of progressing to tuberculosis disease within the first 2 years of their initial exposure,^3,7–9^ and this risk is increased when re-exposed to a recurrent case.

Recurrent tuberculosis accounts for 5–7% of reported tuberculosis cases worldwide, and is particularly common in India.^1,10^ The most recent prevalence survey in India (2019–21) reported that over a quarter of tuberculosis detected was recurrent.^11^ Cohort studies have also demonstrated frequent tuberculosis recurrence in India.^12^ A large multicentre study from India showed that 13% of people with pulmonary tuberculosis who completed treatment developed recurrence within 1 year, for a weighted recurrence rate of 12·7 per 100 person-years.^13^ These data suggest that routine follow-up after treatment completion might be an efficient mechanism for active case finding (ACF) in high-tuberculosis burden settings such as India.

Mathematical models have shown that targeting case finding among tuberculosis survivors could have a substantial effect on transmission.^14^ However, effective strategies for post-tuberculosis screening remain untested. In India, symptom-based screening is recommended at 6-month intervals for 2 years after treatment completion for tuberculosis survivors.^15^ The delivery method for this post-tuberculosis screening intervention has not been defined and household contacts are not recommended for screening in the post-treatment period. When the post-tuberculosis screening recommendation was introduced in India, policy makers considered phone screening (ie, voice calls) as a less resource-intensive alternative to home-based screening but had no supporting evidence (Mave V and Golub JE, Johns Hopkins University, personal communication). We conducted an individually randomised trial to determine whether phone-based screening is non-inferior to home-based screening for detecting tuberculosis in the post-treatment period among tuberculosis survivors and their household contacts.

## Methods

### Study design and participants

TB Aftermath is a pragmatic, hybrid type 1 effectiveness–implementation study^16^ focused primarily on the clinical effectiveness of the systematic screening for tuberculosis intervention in the post-treatment period comparing two implementation strategies: phone-based screening and home-based screening.^17^ Here, we report the primary effectiveness results from the study’s open-label, randomised, controlled, non-inferiority trial in terms of the rate at which tuberculosis was detected among participants receiving each strategy. TB Aftermath’s secondary implementation outcomes and cost-effectiveness results will be reported elsewhere.

TB Aftermath recruited tuberculosis survivors from three urban and three rural tuberculosis clinics under India’s National Tuberculosis Elimination Program (NTEP) in Pune, Maharashtra. Clinics with the largest caseloads were selected. Maharashtra is one of the states in India with the highest tuberculosis burden, with a notification rate of 176 per 100 000 population.^18^ In urban clinics, ACF activities are usually led by a designated home visitor whereas in rural clinics, ACF responsibilities are shared among clinical and laboratory staff.

As a pragmatic trial, TB Aftermath used broad inclusion criteria. We enrolled survivors (aged ≥18 years) within 60 days of treatment completion for any form of tuberculosis. To recruit participants, we reviewed registries at participating clinics monthly, attempted to contact all adults nearing the end of their treatment, and invited interested individuals to the clinic for informed consent. Household contacts (all ages) were enumerated at the survivor’s enrolment visit. Survivors were not required to have household contacts to enrol in the trial.

TB Aftermath was registered with ClinicalTrials.gov (NCT04333485) and the Clinical Trials Registry of India (CTRI/2020/05/025059) in 2020, and approved by the Institutional Review Board at Johns Hopkins School of Medicine, Baltimore, MD, USA (IRB00247239) and Dr D Y Patil University, Pune, India (DYPV/EC/508/2020). Any protocol deviations were reported to both Institutional Review Boards (appendix 1 p 3). Written informed consent was obtained in person from all tuberculosis survivors before enrolment and included permission to enumerate and collect data about household contacts. Enumerated contacts were approached during study visits for written informed consent or if younger than 18 years, parent consent with assent (written if aged ≥12 years, verbal if aged <12 years). Since the intervention is non-invasive and involves minimal risks, adverse events were not anticipated or recorded, and a Data and Safety Monitoring Board was not convened.

### Randomisation and masking

The unit of randomisation was the tuberculosis survivor. After informed consent, survivors were randomly assigned (1:1) to receive phone-based screening or home-based screening. Allocation was computer-generated using permuted blocks of size eight, with stratification by clinic, and placed in sealed envelopes by data managers. After randomisation, participants and study staff were unblinded. This was an open-label trial due to the interactive nature of the intervention. Household contacts were assigned to the same group as the enrolled survivor.

### Procedures

At enrolment, study staff counselled survivors about the risks of tuberculosis recurrence, risks of tuberculosis among their household contacts, and seeking care for tuberculosis symptoms. Detailed clinical and sociodemographic information, as well as point-of-care spirometer measurements were collected from the survivor at baseline. Data on sex were self-reported. The survivor also reported sociodemographic information and history of tuberculosis preventive treatment (TPT) for each of their household contacts. TPT history was collected for contacts younger than 6 years throughout the study; however, in 2022, participating clinics began offering TPT to all household contacts (irrespective of age) and subsequently TPT history was collected for those aged 6 years and older from this point onwards.^19^

Both forms of screening were scheduled 6 months and 12 months after the survivor’s treatment completion date. Symptom screening was conducted by staff from tuberculosis clinics under India’s NTEP to emulate programmatic conditions. All staff received refresher training on the standardised symptom screening questionnaire, which was developed based on WHO and NTEP guidelines and included cough, haemoptysis, fever, weight loss, night sweats, and symptoms of extrapulmonary tuberculosis (eg, lymph node swelling).^2,15^ Study staff screened participants at 6 months and 12 months only if NTEP staff were unable to do so within 45 days of the scheduled screening date.

Survivors in both groups reported their own symptoms at each visit. For enumerated contacts, symptom reporting differed by group: at 6 months and 12 months, symptoms were reported by the survivor in the phone-based screening group but directly by the contact in the home-based screening group if present during the visit. In both groups, household members with any symptom of tuberculosis were referred to the tuberculosis clinic for sputum collection and evaluation by NTEP clinicians per Indian guidelines.^15^ Participants who were potentially eligible for TPT were referred for evaluation.

A minimum of three attempts were made for each scheduled visit before considering an enrolled survivor untraceable. To prevent crossovers between groups, visit attempts were made according to the survivor’s allocated strategy. Thus, survivors who moved out of the study area could continue to be screened only if they were allocated to the phone-based screening group. The TB Aftermath protocol includes a more detailed description of procedures.^17^

### Outcomes

Our predefined primary outcome was the difference in the rate of tuberculosis detection per 100 person-years by group among tuberculosis survivors and their household contacts within 12 months of tuberculosis treatment completion. Our predefined secondary outcome (described in appendix 1 [p 3]) is reported elsewhere.^20^ All tuberculosis diagnoses among survivors or contacts that occurred during this 12-month period were included, regardless of type of diagnosis (microbiologically confirmed or clinically diagnosed, pulmonary and/or extrapulmonary). To characterise tuberculosis events, we administered an in-person questionnaire to household members who were diagnosed with tuberculosis during the 12-month study period and conducted clinical record abstraction to confirm the date and type of tuberculosis diagnosis.

Procedures for outcome ascertainment were the same in each group. To ensure capture of tuberculosis diagnoses that occurred before our phone calls or home visits, we confirmed the tuberculosis history of all household members at the 6-month and 12-month study visits. Additionally, for both groups, we conducted a final home visit at least 18 months after treatment, in which we asked about any tuberculosis diagnoses that occurred during the 12-month study period. Throughout the study, we regularly cross-matched names of enrolled survivors and their enumerated contacts with tuberculosis registries at the study clinics.

### Statistical analysis

Based on previous studies from India,^12,13,21^ we estimated the rate of tuberculosis detection would be 12 per 100 person-years among tuberculosis survivors and 1 per 100 person-years among household contacts, for a weighted mean rate of 4·67 per 100 person-years assuming an average of two contacts per survivor. The non-inferiority margin for our primary outcome (survivors and contacts combined) was set a priori as a rate difference of 1·70 per 100 person-years, calculated as the rate in the home-based screening group minus the rate in the phone-based screening group (details are specified in appendix 1 [p 3]). Non-inferiority was established when the upper bound of the 95% CI around the rate difference was less than 1·70 per 100 person-years, corresponding with a one-sided alpha level of 0·025. Our hypothesis was that phone-based screening would be non-inferior to home-based screening. A sample size of 1076 survivors and 2152 contacts was calculated to provide 90% power that phone screening was non-inferior to home-based screening (within the 1·70 per 100 person-years margin, assuming true difference of zero).

For our prespecified primary analysis, we assumed a Poisson distribution to calculate detection rates and adjusted for household-level clustering of tuberculosis by fitting a multilevel Poisson model with household-level random effects and log-person-time as an offset.^22^ To calculate 95% CIs, we assumed a Poisson distribution for detection rates and a normal distribution for rate differences.

Our primary analysis was intention-to-treat and our post-hoc secondary analysis used a per-protocol approach. In our intention-to-treat population, we included all household members, even if they never received the post-tuberculosis screening intervention. However, we were also interested in examining whether phone-based screening was non-inferior to home-based screening when everyone received both the 6-month and 12-month screening visit as planned (ie, with higher fidelity). Thus, the per-protocol population excluded household members lost to follow-up before receiving any post-tuberculosis screening, and those only screened once (at 6 months or 12 months in the post-treatment period). Additionally, the per-protocol population excluded people diagnosed with tuberculosis before the 6-month screening visit and who died during the study period. People with tuberculosis detected between the 6-month and 12-month post-tuberculosis visits remained in the per-protocol population.

The per-protocol population captures tuberculosis events most likely to be actively detected by a TB Aftermath visit (as opposed to passively detected). Thus, we assessed screening yield in this population by number needed to screen (NNS) to detect one case. NNS is calculated as the inverse of the proportion of tuberculosis detected among those screened. Lower NNS indicates higher screening yield.

Person-time at risk was calculated as the time from the survivor’s enrolment to the date of tuberculosis diagnosis, death (all-cause), loss to follow-up, or administrative censoring. We approximated loss to follow-up dates based on when the participant moved out of the area, was considered untraceable after three visit attempts, or refused further screening. Person-time included a maximum of 15 months to capture the 3-month period around the 12-month visit. We performed a prespecified subgroup analysis by participant type (survivor *vs* contact) for which we directly compared tuberculosis detection rates by screening strategy within each subgroup (evaluated on a superiority hypothesis using a two-sided Wald test for statistical significance). Among survivors, we assessed crude rates and stratified by treatment history (treated for pulmonary disease *vs* only extrapulmonary disease). We made group-wise comparisons of median months to diagnosis using the cluster-adjusted Wilcoxon rank sum test, and of TPT history using the cluster-adjusted Rao-Scott χ^2^ test.

We conducted four post-hoc sensitivity analyses to assess the robustness of our findings: we performed Cox proportional hazards regression instead of Poisson regression and used Kaplan–Meier plots with cluster-robust standard errors to assess equality of survival functions; we reclassified confirmed or probable tuberculosis-related deaths among survivors as recurrences, based on verbal autopsies conducted by study staff; we restricted our analyses to include microbiologically confirmed tuberculosis events only (ie positive by sputum culture, smear microscopy, or rapid molecular testing [ie, cartridge-based nucleic acid amplification test]) based on NTEP records;^15^ and we modified our per-protocol population to include participants who received at least one screening visit. To collect verbal autopsy data, we followed WHO standards and used their Open Data Kit for verbal autopsy.^23^ We evaluated the results of these sensitivity analyses under a superiority hypothesis; the prespecified non-inferiority margin was only applied to the primary analysis.

For all analyses, we used R version 4.1.3 (tidyverse, fmsb, and lme4). For comparisons including contacts, we used cluster-robust standard errors. Across analyses, except the primary non-inferiority analysis, we considered a p value of less than 0·05 to be statistically significant.

### Role of the funding source

The funder had no role in study design, data collection, data analysis, data interpretation, or writing of the report.

## Results

Between Jan 21, 2021, and Sept 4, 2023, we approached 1310 tuberculosis survivors and enrolled 1076 (82%) within 60 days of their tuberculosis treatment completion. Among the enrolled tuberculosis survivors, 537 (50%) were randomly assigned to phone-based screening and 539 (50%) to home-based screening after tuberculosis treatment. In the phone-based screening group, 1638 household contacts were enumerated, and in the home-based screening group, 1671 household contacts were enumerated. On average, approximately three household contacts were enumerated per survivor (mean 3·1 [SD 1·8]). Overall, 2210 tuberculosis survivors and household contacts from the phone-based screening group and 2175 tuberculosis survivors and contacts from the home-based screening group were included in the intention-to-treat population (figure 1).

Sociodemographic characteristics were similar among tuberculosis survivors by group and among household contacts by group. Among 1076 tuberculosis survivors, median age was 35 years (IQR 27–49), 507 (47%) were female, and 569 (53%) were male. Among 3309 enumerated household contacts, median age was 26 years (IQR 13–43), 1648 (50%) were female, and 1661 (50%) were male. One-third of household contacts (1060 [33%] household contacts) were younger than 18 years (table 1). Among 1288 household contacts who reported TPT history, 604 (47%) had taken TPT: 265 (43%) in the phone-based screening group and 339 (50%) in the home-based screening group (p=0·16; appendix 1 p 4).

Clinical characteristics among tuberculosis survivors were also similar by group. More than one-third (371 [34%]) of tuberculosis survivors were underweight or severely underweight (BMI <18·5 kg/m^2^) and ten (1%) were living with HIV. Overall, 148 (14%) tuberculosis survivors were living with diabetes, among whom 59 (81%) were on treatment in the home-based screening group and 50 (69%) were in the phone-based screening group (p=0·30). Across groups, 110 (11%) tuberculosis survivors reported unhealthy alcohol use and 96 (9%) had a history of smoking. Based on point-of-care spirometer measurements, 250 (23%) tuberculosis survivors had signs of lung impairment with a low peak expiratory flow (<200 L per min). At enrolment, 674 (63%) tuberculosis survivors were treated for pulmonary tuberculosis, 35 (3%) were treated for drug-resistant tuberculosis, and 87 (8%) had more than one previous episode of tuberculosis (table 2).

Among 4385 participants, 105 (2%) tuberculosis events were detected: 41 in the phone-based screening group and 64 in the home-based screening group. Among 1076 tuberculosis survivors, 75 (7%) had tuberculosis recurrence detected: 27 in the phone-based screening group and 48 in the home-based screening group. Among 3309 household contacts, 30 (1%) had tuberculosis detected: 14 in the phone-based screening group and 16 in the home-based screening group. The median time to tuberculosis diagnosis among the 105 tuberculosis events was 5·6 months (IQR 3·4–10·6): 5·4 months (3·1–9·6) among tuberculosis survivors and 6·1 months (3·9–10·8) among contacts. Median time to tuberculosis diagnosis was not statistically significantly different between groups (appendix 1 p 6). Across both groups, 26 (1%) participants died without a documented tuberculosis event (22 tuberculosis survivors and four household contacts; figure 1).

Over a median follow-up time of 14·6 months (IQR 13·9–14·9), the crude rate of tuberculosis detection was 2·05 per 100 person-years (95% CI 1·67–2·48) across both groups. We observed clustering of tuberculosis diagnoses at the household level, with similar patterns by group (appendix 1 p 3). After adjusting for clustering, the tuberculosis detection rate per 100 person-years was 1·19 (95% CI 0·82–1·71) in the phone-based screening group and 1·92 (1·37–2·45) in the home-based screening group. For our primary outcome, prespecified non-inferiority was reached with a rate difference of 0·73 (95% CI 0·05–1·41; table 3).

Among the 75 tuberculosis survivors with recurrence detected, 17 (23%) were among extrapulmonary tuberculosis survivors, four (24%) of whom went on to develop pulmonary tuberculosis (appendix 1 p 7). The overall rate of recurrence detection among survivors was 6·15 per 100 person-years (95% CI 4·84–7·71): 7·40 (5·62–9·56) among pulmonary tuberculosis survivors and 3·90 (2·27–6·25) among extrapulmonary tuberculosis survivors. The recurrence detection rate per 100 person-years was statistically significantly lower in the phone-based screening group (4·36 [95% CI 2·87–6·34] *vs* the home-based screening group (8·00 [5·90–10·60]; rate difference 3·64 [95% CI 0·84–6·44]; p=0·01; table 3; figure 2A). Recurrence detection rates were also lower in the phone-based screening group compared with the home-based screening group in the stratified analysis of pulmonary tuberculosis survivors, but not in the extrapulmonary tuberculosis survivors (table 3).

Among the 30 (1%) household contacts with tuberculosis detected, six (20%) were children (<18 years) and eight (27%) developed extrapulmonary tuberculosis without pulmonary involvement. Most (26 [83%]) of these tuberculosis diagnoses were among household contacts living with survivors of pulmonary tuberculosis (appendix 1 p 7). The cluster-adjusted tuberculosis detection rate per 100 person-years among contacts was 0·61 (95% CI 0·32–1·07) in the phone-based screening group and 0·70 (0·39–1·20) in the home-based screening group. These rates were not statistically significantly different (p=0·70; table 3; figure 2A).

For the per-protocol population, 241 (22%) tuberculosis survivors and 1328 (40%) household contacts met exclusion criteria. Most (1281 [82%]) exclusions were in the home-based screening group, which reached fewer participants with screening visits compared with the phone-based screening group (figure 1). Sociodemographic characteristics of participants in the per-protocol population remained similar by group except regarding the rural versus urban setting: fewer people in rural settings were reached by both the 6-month and 12-month home-based screening visits (appendix 1 p 8). After these exclusions, non-inferiority criterion was still met in the per-protocol population based on a rate difference of 0·85 (95% CI 0·07–1·62). Consistent with the intention-to-treat analysis, recurrence detection rate among tuberculosis survivors remained lower in the phone-based screening group compared with the home-based screening group (rate difference 2·09 per 100 person-years [0·00–4·18]; p=0·05). Tuberculosis detection remained similar by group for household contacts in the per-protocol population (p=0·92; figure 2B; table 3).

Among the 105 total tuberculosis events, 35 (33%) were included in the per-protocol analysis. Of the 1887 participants in the per-protocol population who received phone-based screening, 16 (1%) tuberculosis events were detected for an NNS of 118. Of the 929 participants in the per-protocol population who received home-based screening, 19 (2%) tuberculosis events were detected for an NNS of 49. Overall, and among subgroups, NNS was consistently higher in the phone-based screening group compared with the home-based screening group (appendix 1 p 9).

In our first sensitivity analysis using Cox proportional hazards, home-based screening was more effective than phone-based screening for detecting tuberculosis in the combined analysis and in the tuberculosis survivor subgroup for both the intention-to-treat and per-protocol populations (figure 3). Consistent with the main analysis, the survival curves for the household contact subgroup were similar by group for both the intention-to-treat and per-protocol populations (appendix 1 p 11). Second, through verbal autopsies, we identified six confirmed and one probable tuberculosis-related deaths with no previous tuberculosis recurrence event recorded. Reclassifying these seven deaths as tuberculosis recurrences did not change our main findings. Third, our sensitivity analysis restricted to microbiologically confirmed tuberculosis events included 30 (73%) of 41 events in the phone-based screening group and 37 (58%) of 64 events in the home-based screening group. The non-inferiority criterion was again met in the intention-to-treat and per-protocol population in this restricted analysis. In the tuberculosis survivor subgroup, the rate of tuberculosis recurrence detection remained lower with phone-based screening compared with home-based screening, but the difference was no longer statistically significant in the intention-to-treat population (p=0·12). Finally, when modifying our per-protocol population to include people reached by one or more visits, results were similar to the main analysis (appendix 1 p 10).

## Discussion

To our knowledge, the TB Aftermath study from India represents the first published individually randomised trial on screening for tuberculosis in the post-treatment period. We found phone-based screening was non-inferior to home-based screening for detecting tuberculosis among survivors and their household contacts in the 1-year period after treatment. For detection of tuberculosis among household contacts specifically, tuberculosis rates were also similar by group. However, screening for tuberculosis at people’s homes was a more effective strategy for detecting recurrence among survivors. In our primary analysis, the rate of tuberculosis recurrence detection was nearly double in the home-based screening group and statistically significantly different from the phone-based screening group. The comparative effectiveness of home-based screening for detecting recurrence was evident across analyses, regardless of exclusions or model specification.

In the per-protocol analysis, which only included people who received the intervention, the non-inferiority margin was also met but the difference between groups was more pronounced than in our intention-to-treat analysis. This change was driven by the higher number of recurrences detected through home visits, despite fewer participants in that group completing visits; it was not feasible to visit homes outside of the study area. Additionally, because rural clinics have fewer staff dedicated to outreach and rural homes are harder to reach, home visits were less likely to be completed for rural participants. By contrast, we did not encounter issues following up tuberculosis survivors by phone, due in part to high phone ownership in urban and rural India.^24^ Overcoming logistical barriers to implementing home visits, including in rural areas, might enhance effectiveness of post-tuberculosis screening. Finally, similar to the primary results, the difference in detection rates among contacts in the per-protocol analysis was negligible. However, per-protocol results should be interpreted cautiously because the participants included in this analysis might not be representative of the broader study population, and associations of tuberculosis risk with urban residence or spending more time at home could confound results.

Evidence from our pragmatic trial highlights the substantial risk of tuberculosis among households in the post-treatment period, although our observed rates were slightly lower than previous data from India.^12,13,21^ Among household contacts, the rate per 100 person-years was below 1 in both groups in our primary analysis and in the per-protocol analysis. Our household contact detection rate was likely lower than previous studies because: previous studies screened closer to index patient diagnoses when risk of disease progression might be higher;^9^ we adjusted for household-level clustering, which attenuated rates compared with crude estimates; and nearly half of the contacts in our study had previously taken TPT, which was likely higher than previous studies due to India’s continued scale-up of TPT.^18^ The overall tuberculosis recurrence rate that we observed (6·15 per 100 person-years) was also lower than previous estimates, but this rate was high compared with general-population incidence and even compared with other high-risk groups such as people living with HIV in India (estimated incidence 1·42 per 100 person-years).^25,26^ The lower rate of recurrence relative to pre-trial data might be explained by India’s shift from a thrice-weekly to a daily tuberculosis regimen, which has been shown to improve treatment outcomes.^27^

Before this study, recurrence among extrapulmonary tuberculosis survivors had not been well characterised. Over one-third of tuberculosis survivors enrolled in TB Aftermath were treated for extrapulmonary disease only, reflecting India’s estimated burden of extrapulmonary disease.^18^ Without any documentation of pulmonary involvement, this group had a tuberculosis recurrence rate of nearly 4 per 100 person-years. TB Aftermath data reported elsewhere showed that people treated for extrapulmonary tuberculosis also had substantial lung impairment.^28^ Together, these results support the need for post-tuberculosis interventions targeting all tuberculosis survivors, regardless of history of pulmonary involvement. Follow-up in person (as opposed to telephonically) has the added benefit of being able to offer more comprehensive care for post-tuberculosis sequelae.

Our prespecified primary trial endpoint included both passively and actively detected tuberculosis. We also explored the direct yield of each screening strategy for actively detecting tuberculosis and estimated a low NNS among tuberculosis survivors compared with data reported elsewhere for high-risk groups. Specifically, a recent review of ACF interventions in India reported a weighted mean NNS of 50 across 12 studies of household contacts investigations conducted shortly after index patient diagnosis.^29^ While we observed a higher NNS for household contact investigation in the post-treatment period (>180), it is noteworthy that the NNS among survivors screened at home was only 23 and it was 52 for those screened by phone. Our findings are consistent with the Targeted Universal Testing for Tuberculosis (TUTT) study in clinical settings in South Africa which also demonstrated that ACF among tuberculosis survivors was high yield—TUTT detected tuberculosis among 12% of people with recent previous tuberculosis based on Xpert MTB/RIF Ultra or culture positivity.^30^

Our main objective was to evaluate two different delivery methods for ACF in the post-treatment period. Thus, in both groups, our intervention aligned with current guidelines in India for the timing of post-treatment screening (6-month intervals). However, we estimated that our intervention only actively detected about one-third of tuberculosis events (35 of 105) over a 1-year period, and the median time to diagnosis was less than 6 months after treatment (ie, before any screening occurred). Given these findings, we hypothesise that earlier screening timepoints would increase the yield of post-tuberculosis ACF. Additionally, the advantages of home-based screening might be even more pronounced if implemented sooner after treatment completion. Further research is needed to explore this hypothesis.

Our trial addresses a major evidence gap around effective implementation strategies for post-treatment screening. However, our study has several limitations. First, although we reached our target sample size, our trial was not powered to test the difference between groups for tuberculosis survivors and contacts separately nor for the secondary per-protocol analysis. To mitigate this limitation, we conducted several sensitivity analyses and showed that home-based screening was consistently better than phone screening for detecting recurrences. Additionally, although we adjusted for household-level clustering, we did not account for this in our sample size calculations. However, only seven households had more than one tuberculosis event. Second, survivors of drug-resistant tuberculosis and those living with HIV are under-represented. Additionally, only one district is included in this study. However, by recruiting from public clinics in both rural and urban areas in a state of India with a high burden of tuberculosis, we believe that our results could be generalisable to other similar high-burden settings for screening among people treated for drug-susceptible tuberculosis. Third, phone screening asked tuberculosis survivors to report on household contacts’ symptoms and home visits did not evaluate contacts who were not home. These limitations likely attenuated the detection rate among household contacts, although both reflect realities of pragmatic household-level screening. Additionally, although people who moved out of the area were considered lost to follow-up in the home-based screening group (n=22), screening was done for those who moved out of the area in the phone-based screening group (n=43), which could have artificially inflated tuberculosis detection in the phone-based screening group. Fourth, to align with current Indian guidelines, we relied on symptom screening for ACF, which has suboptimal performance.^2^ This screening method might have resulted in lower case detection but with non-differential bias by group. Future studies should explore the yield of post-treatment screening using point-of-care tools with higher sensitivity and specificity.

Risk of tuberculosis remains high for households with a tuberculosis survivor. For detecting tuberculosis among survivors and their household contacts together, phone-based screening was non-inferior to home-based screening in the post-treatment period. However, for detecting tuberculosis recurrence, visiting the homes of tuberculosis survivors was more effective than screening them over the phone. Although resource trade-offs still need to be evaluated, countries with a high burden of tuberculosis recurrence should consider home-based strategies beginning <6 months after treatment for post-tuberculosis screening to help close the case-detection gap.

## Supplementary Material

1

2

## Figures and Tables

**Figure 1:**
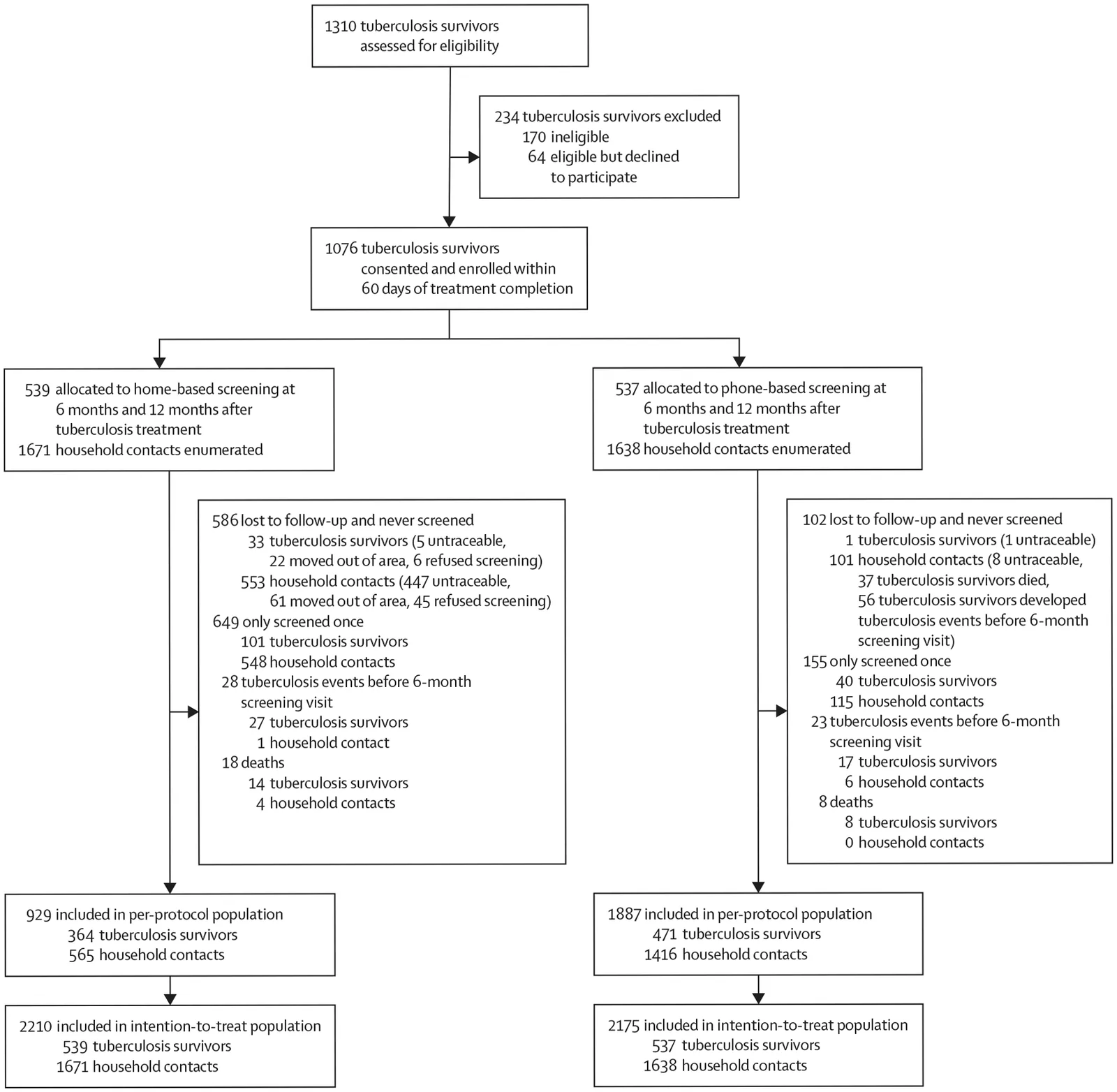
Trial profile

**Figure 2:**
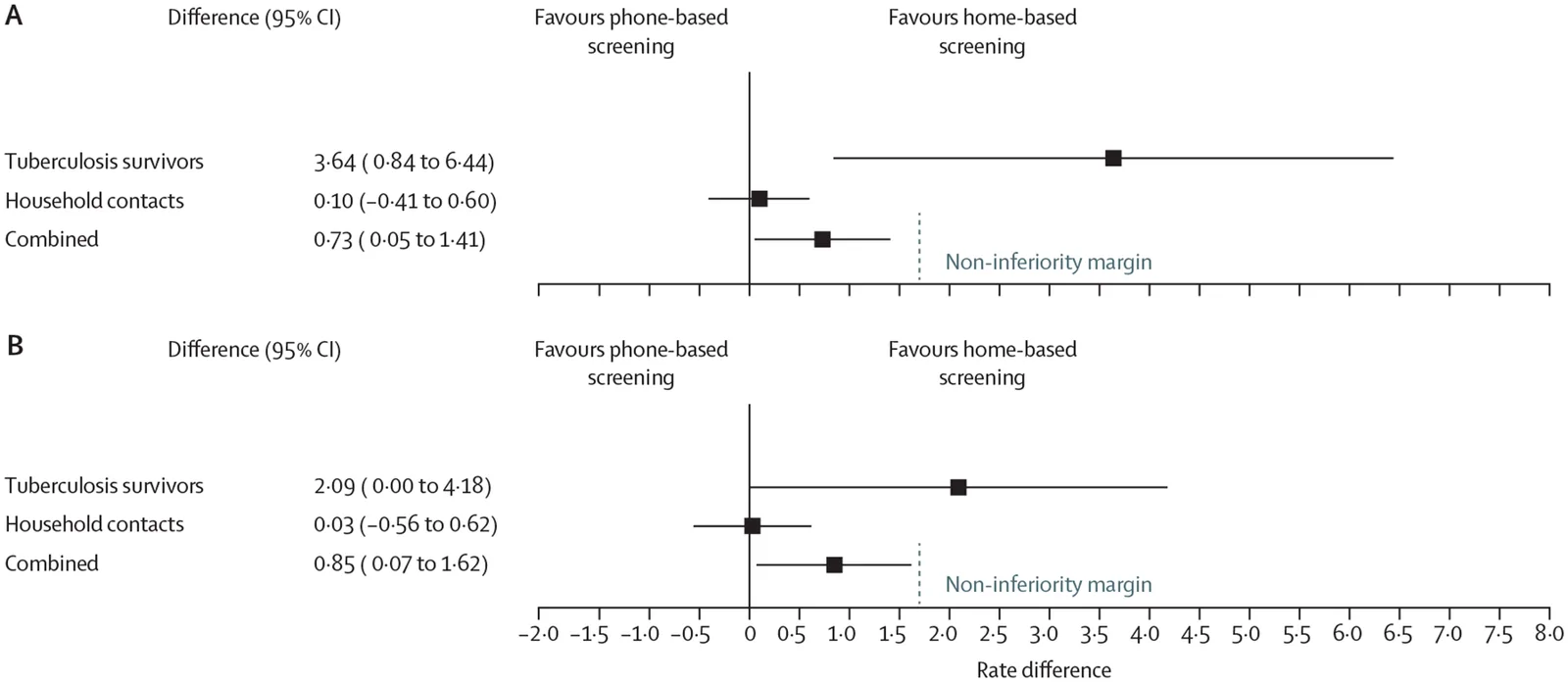
Tuberculosis detection rate within 12 months in the post-treatment period (A) Intention-to-treat population including all household members, including those who never received the post-tuberculosis screening intervention. (B) Per-protocol population excluding household members who were lost to follow-up before receiving any post-tuberculosis screening, and those who were only screened once.

**Figure 3:**
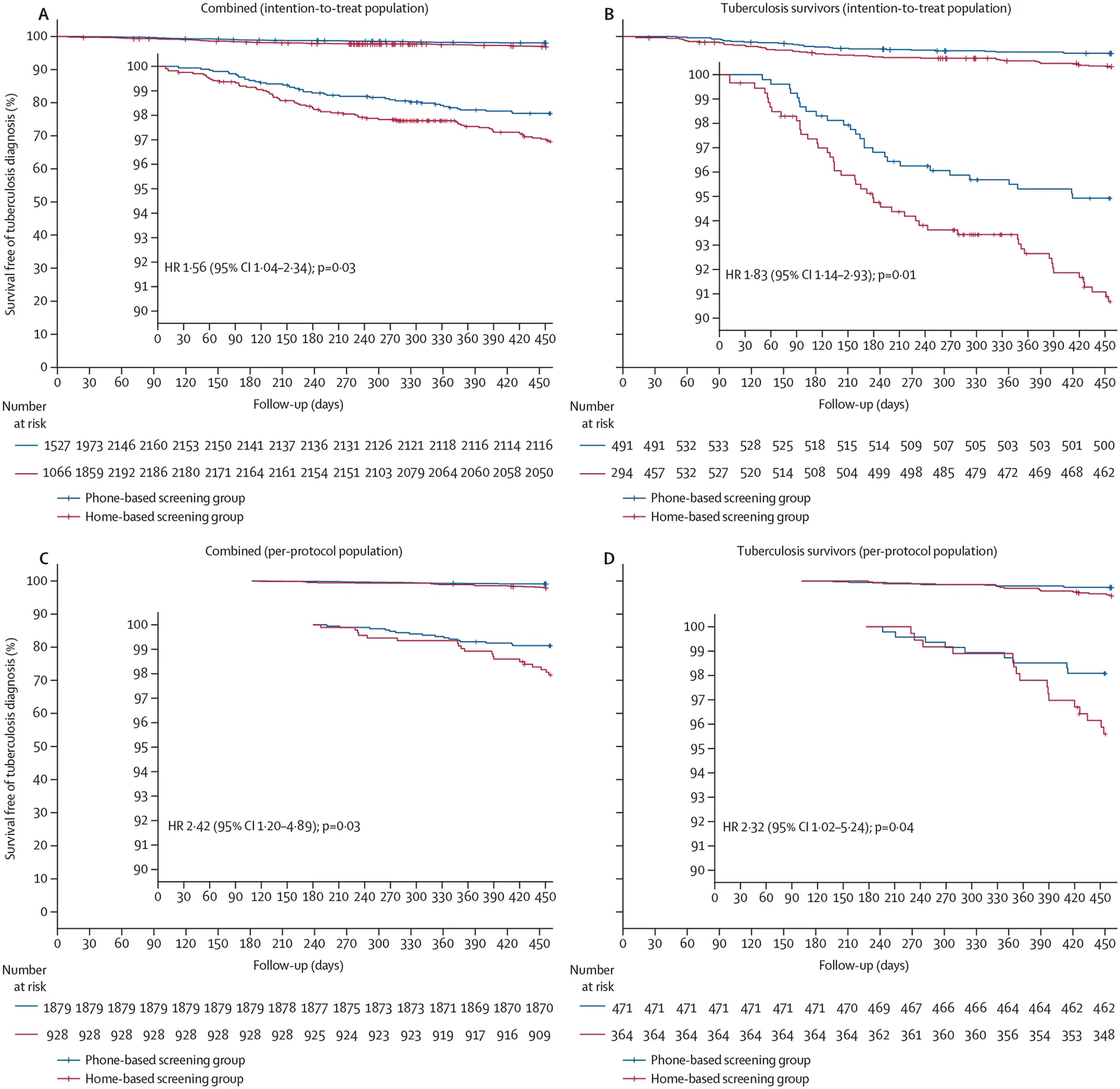
Survival free of tuberculosis diagnosis by group p value is calculated based on the robust log-rank test comparing survival free of tuberculosis diagnosis in the home-based screening group compared with the phone-based screening group. (A) Tuberculosis survivors and household contacts combined (intention-to-treat population). (B) Tuberculosis survivors only (intention-to-treat population). (C) Tuberculosis survivors and household contacts combined (per-protocol population). (D) Tuberculosis survivors only (per-protocol population). HR=hazard ratio.

**Table 1:** Sociodemographic characteristics of tuberculosis survivors and household contacts (intention-to-treat population)

	Tuberculosis survivors			Household contacts		
	Home-based screening group (n=539)	Phone-based screening group (n=537)	All (n=1076)	Home-based screening group (n=1671)	Phone-based screening group (n=1638)	All (n=3309)
Age, years	35 (26–49)	35 (27–49)	35 (27–49)	26 (13–45)	26 (14–42)	26 (13–43)
Age group						
<6 years	NA	NA	NA	182 (11%)	160 (10%)	342 (10%)
6–17 years	NA	NA	NA	360 (22%)	358 (22%)	718 (22%)
18–24 years	97 (18%)	103 (19%)	200 (19%)	229 (14%)	239 (15%)	468 (14%)
25–49 years	313 (58%)	303 (56%)	616 (57%)	597 (36%)	605 (37%)	1202 (36%)
≥50 years	129 (24%)	131 (24%)	260 (24%)	303 (18%)	276 (17%)	579 (17%)
Sex*						
Female	246 (46%)	261 (49%)	507 (47%)	832 (50%)	816 (50%)	1648 (50%)
Male	293 (54%)	276 (51%)	569 (53%)	839 (50%)	822 (50%)	1661 (50%)
Setting						
Rural	212 (39%)	210 (39%)	422 (39%)	607 (36%)	570 (35%)	1177 (36%)
Urban	327 (61%)	327 (61%)	654 (61%)	1064 (64%)	1068 (65%)	2132 (64%)
Highest education attained†						
Illiterate or no formal education	82 (15%)	68 (13%)	150 (14%)	200/1498 (13%)	209/1478 (14%)	409/2967 (14%)
Primary school	188 (35%)	194 (36%)	382 (36%)	622/1498 (42%)	599/1478 (41%)	1221/2967 (41%)
Secondary or high school	157 (29%)	164 (31%)	321 (30%)	442/1498 (30%)	432/1478 (29%)	874/2967 (29%)
University or graduate school	110 (20%)	111 (21%)	221 (21%)	220/1498 (15%)	229/1478 (15%)	449/2967 (15%)
Biomass exposure‡	142 (26%)	133 (25%)	275 (26%)	454 (27%)	422 (26%)	876 (26%)
Household income, Indian rupees per month						
≤5000	24 (5%)	35 (7%)	59 (5%)	41 (2%)	53 (3%)	94 (3%)
5001–10000	74 (14%)	86 (16%)	160 (15%)	184 (11%)	223 (14%)	407 (12%)
>10 000	439 (81%)	414 (77%)	853 (79%)	1439 (86%)	1356 (83%)	2795 (84%)
Employed (among adults aged ≥18 years)§	291 (54%)	271 (50%)	562 (52%)	619/1129 (55%)	616/1120 (55%)	1235/2249 (55%)

Data are n (%), n/N (%), or median (IQR). Percentages might not add to 100 due to rounding. Column-wise proportions are provided. Missingness: <1% missing data for each group: highest education attained (16 of 4043 individuals aged >6 years); household income (17 of 4385 participants). NA=not applicable.

* Reflects self-reported biological sex.

† Children aged <6 years excluded from denominator (n=182 for home-based screening group and n=160 for phone-based screening group).

‡ Includes primary or secondary exposure to unclean cooking fuel, such as kerosene or wood. Of 275 tuberculosis survivors with biomass exposure, 202 (73%) were from rural areas (100 [70%] of 142 tuberculosis survivors in the home-based screening group and 102 [77%] of 133 tuberculosis survivors in the phone-based screening group). Of 876 household contacts with biomass exposure, 610 (70%) were from rural areas (311 [69%] of 454 household contacts in the home-based screening group and 299 [71%] of 422 household contacts in the phone-based screening group).

§ Employed was defined as having at least part-time work (formal or informal contract). Denominator includes 1129 household contacts aged ≥18 years from the home-based screening group and 1120 from the phone-based screening group.

**Table 2:** Clinical characteristics of tuberculosis survivors (intention-to-treat population)

	Home-based screening group (n=539)	Phone-based screening group (n=537)	All tuberculosis survivors (n=1076)
BMI, kg/m^2^	19⋅7 (17⋅4–22⋅8)	20⋅1 (17⋅7–23⋅2)	19⋅9 (17⋅5–23⋅1)
BMI categories, kg/m^2^			
<16⋅5 (severely underweight)	96 (18%)	78 (15%)	174 (16%)
16⋅5–18⋅4 (underweight)	98 (18%)	99 (18%)	197 (18%)
18⋅5–24⋅9 (healthy weight)	274 (51%)	283 (53%)	557 (52%)
≥25⋅0 (overweight)	71 (13%)	77 (14%)	148 (14%)
HIV			
Yes*	4 (1%)	6 (1%)	10 (1%)
No	514 (95%)	507 (94%)	1021 (95%)
Unknown	21 (4%)	24 (5%)	45 (4%)
Hypertension†	39 (7%)	39 (7%)	78 (7%)
On treatment	29 (74%)	28 (72%)	57 (73%)
Diabetes‡	76 (14%)	72 (13%)	148 (14%)
On treatment	59 (78%)	50 (69%)	109 (75%)
Coronary heart disease†	9 (2%)	6 (1%)	15 (1%)
On treatment	9 (100%)	6 (100%)	15 (100%)
Unhealthy alcohol use§			
Harmful or hazardous use	38 (7%)	22 (4%)	60 (6%)
Alcohol dependence	27 (5%)	23 (4%)	50 (5%)
History of smoking^¶^			
Current	22 (4%)	18 (3%)	40 (4%)
Past	29 (5%)	27 (5%)	56 (5%)
Peak expiratory flow, L per min||	273 (194–371)	279 (192–356)	278 (193–361)
Peak expiratory flow categories, L per min||			
Low	127 (24%)	123 (23%)	250 (23%)
Medium	200 (37%)	193 (36%)	393 (37%)
High	132 (24%)	126 (23%)	258 (24%)
Site of disease in previous tuberculosis episode			
Extrapulmonary	179 (33%)	197 (37%)	376 (35%)
Pulmonary	344 (64%)	330 (61%)	674 (63%)
Both	16 (3%)	10 (2%)	26 (2%)
Microbiological confirmation of cure among participants with pulmonary involvement in previous tuberculosis episode	357/360 (99%)	335/340 (99%)	692/700 (99%)
>1 previous episode of tuberculosis	39 (7%)	48 (9%)	87 (8%)
Drug resistance in previous tuberculosis episode	19 (4%)	16 (3%)	35 (3%)

Data are median (IQR), n/N (%), or n (%). Percentages might not add to 100 due to rounding provided. Column-wise proportions are provided. Missingness: peak expiratory flow, 175 (15%) of 1076 participants overall; 80 (15%) of 539 participants in the home-based screening group and 95 (18%) of 537 participants in the phone-based screening group. For all other variables, <1% missing data for each group: coronary heart disease (one of 1076 participants); unhealthy alcohol use (one of 1076 participants). AUDIT=alcohol use disorders identification test.

* All tuberculosis survivors known to be living with HIV (ten [1%] of 1076 participants) reported being on antiretroviral therapy.

† Hypertension and coronary heart disease were self-reported for all tuberculosis survivors.

‡ Diabetes is defined as previously diagnosed, self-reported diabetes, or HbA_1c_ ≥6·5%.

§ Alcohol dependence is defined as an AUDIT score of >13 for female participants and >15 for male participants. Harmful or hazardous use is defined as an AUDIT score of 8–12 for female participants and 8–14 for male participants. Of 507 female tuberculosis survivors, only one reported any alcohol use with an AUDIT score <8.

¶ Only one female participant reported being a current smoker. All past smokers were male.

|| Peak expiratory flow was measured during point-of-care spirometry assessments for a subset of participants. Low peak expiratory flow is defined as <200 L per min, medium peak expiratory flow is defined as 200–299 L per min for female participants and 200–399 L per min for male participants, and high peak expiratory flow is defined as >300 L per min for female participants and >400 L per min for male participants.

**Table 3:** Rate of tuberculosis detection among tuberculosis survivors and household contacts by study group

	Number of participants in the home-based screening group	Events per person-years in the home-based screening group	Detection rate per 100 person-years* in the home-based screening group	Number of participants in the phone-based screening group	Events per person-years in the phone-based screening group	Detection rate per 100 person-years* in the phone-based screening group	Detection rate difference (95% CI)†
Combined (tuberculosis survivors and household contacts)‡							
Intention-to-treat	2210	64/2562	1⋅92 (1⋅37 to 2⋅45)	2175	41/2568	1⋅19 (0⋅82 to 1⋅71)	0⋅73 (0⋅05 to 1⋅41)
Per-protocol	929	19/1108	1⋅45 (0⋅83 to 2⋅34)	1887	16/2255	0⋅60 (0⋅34 to 1⋅04)	0⋅85 (0⋅07 to 1⋅62)
Household contacts							
Intention-to-treat	1671	16/1962	0⋅70 (0⋅39 to 1⋅20)	1638	14/1948	0⋅61 (0⋅32 to 1⋅07)	0⋅10 (−0⋅41 to 0⋅60)
Per-protocol	565	3/675	0⋅44 (0⋅09 to 1⋅30)	1416	7/1692	0⋅41 (0⋅16 to 0⋅85)	0⋅03 (−0⋅56 to 0⋅62)
Tuberculosis survivors							
Intention-to-treat	539	48/600	8⋅00 (5⋅90 to 10⋅60)	537	27/620	4⋅36 (2⋅87 to 6⋅34)	3⋅64 (0⋅84 to 6⋅44)
Per-protocol	364	16/433	3⋅69 (2⋅11 to 6⋅00)	471	9/562	1⋅60 (0⋅73 to 3⋅04)	2⋅09 (0⋅00 to 4⋅18)
Tuberculosis survivors treated for extrapulmonary tuberculosis							
Intention-to-treat	179	10/205	4⋅87 (2⋅33 to 8⋅95)	197	7/230	3⋅04 (1⋅22 to 6⋅26)	1⋅83 (−1⋅94 to 5⋅59)
Per-protocol	132	5/157	3⋅19 (1⋅04 to 7⋅45)	173	3/206	1⋅46 (0⋅30 to 4⋅26)	1⋅73 (−1⋅51 to 4⋅98)
Tuberculosis survivors treated for pulmonary tuberculosis							
Intention-to-treat	360	38/395	9⋅63 (6⋅81 to 13⋅20)	340	20/389	5⋅13 (3⋅14 to 7⋅93)	4⋅49 (0⋅69 to 8⋅29)
Per-protocol	232	11/277	3⋅98 (1⋅99 to 7⋅12)	298	6/357	1⋅68 (0⋅62 to 3⋅66)	2⋅30 (−0⋅41 to 5⋅00)

In our intention-to-treat population, we included all household members, even if they never received the post-tuberculosis screening intervention. The per-protocol population excluded household members who were lost to follow-up before receiving any post-tuberculosis screening, as well as those who were only screened once (either at 6 months or 12 months in the post-treatment period). Additionally, the per-protocol population excluded people who were diagnosed with tuberculosis before the 6-month screening visit and those who died during the study period. People with tuberculosis detected between the 6-month and 12-month post-tuberculosis screening visits remained in the per-protocol population.

* Assumed Poisson distribution to calculate detection rates and group-specific CIs. For tuberculosis survivors and household contacts (combined) and household contacts (alone), we adjusted for household-level clustering.

† 95% CI for detection rate difference calculated using normal approximation.

‡ Primary outcome: tuberculosis rate per 100 person-years by group among tuberculosis survivors and household contacts within 12 months. Non-inferiority is established if the upper bound of the 95% CI around the rate difference was less than 1·70 per 100 person-years.

## Data Availability

De-identified participant data (including the data dictionary) and statistical code can be made available on request to the corresponding author (scox26@jh.edu).

## Research in context

### Evidence before this study

We searched for trials published in PubMed, Embase, and Cochrane, without language restrictions, from Jan 1, 2010, to March 31, 2026, using the terms, and variations of, “tuberculosis” [AND] “screening” [AND] “post-treatment” [OR] “recurrence”. The only published trial that reported on tuberculosis screening in the post-treatment period was the Targeted Universal Testing for Tuberculosis (TUTT) cluster-randomised trial from South Africa. The TUTT trial found that facility-based systematic screening among tuberculosis survivors with a recent tuberculosis episode was high yield; tuberculosis recurrence was detected based on Xpert MTB/RIF Ultra or culture positivity among 12·0% of tuberculosis survivors screened.

### Added value of this study

Our TB Aftermath study from India represents the first individually randomised trial on screening for tuberculosis in the post-treatment period. Before TB Aftermath, there had also not been any trials on implementation strategies for community-based tuberculosis detection after treatment completion. Our pragmatic trial results demonstrate that the risk of tuberculosis remains high for households with a tuberculosis survivor in high-burden settings such as India. We found that for detecting tuberculosis among survivors and their household contacts together, phone-based screening was non-inferior to home-based screening in the post-treatment period. However, screening for tuberculosis at people’s homes was a more effective strategy for detecting tuberculosis recurrence among survivors.

### Implications of all the available evidence

Our findings address a major evidence gap around systematic screening for tuberculosis disease among high-risk groups. In the most recent WHO guidelines on the topic, there was only a conditional recommendation for screening among people with a history of tuberculosis due to insufficient evidence. Our study offers strong evidence that tuberculosis survivors in high-incidence settings such as India are a high-risk group that would benefit from systematic tuberculosis screening. Although resource trade-offs still need to be evaluated, countries with a high burden of tuberculosis recurrence should consider home-based strategies for post-tuberculosis screening to help close the case-detection gap.
