# Supplementary material for: Phone-based screening versus home-based screening after tuberculosis in India (TB Aftermath): a multicentre, open-label, randomised, controlled, non-inferiority trial
Source: Lancet Glob Health. Author manuscript; Available in PMC 2026 Oct 20. (PMC13681456; doi:10.1016/j.langlo.2026.103971)
Supplement: Supplementary file 1 [file NIHMS2208782-supplement-1.pdf]

THE LANCET
Global Health

## Supplementary appendix 1

This appendix formed part of the original submission and has been peer reviewed.
We post it as supplied by the authors.

Supplement to: Cox SR, Kadam A, Valawalkar S, et al. Phone-based screening versus home-based screening after tuberculosis in India (TB Aftermath): a multicentre, open-label, randomised, controlled, non-inferiority trial. *Lancet Glob Health* 2026. https://doi.org/10.1016/j.langlo.2026.103971

**Supplemental material to Phone-based screening versus home-based screening after tuberculosis in India (TB Aftermath): a multicentre, open-label, randomized, controlled, non-inferiority trial**

Table of Contents

TB Aftermath Protocol & Additional Information ........ 3

Similar patterns of household clustering of tuberculosis by group (intention-to-treat population) ........ 3

Table S1: Tuberculosis preventive treatment history among enumerated household contacts (intention-to-treat population) ........ 4

Table S2: Rate of tuberculosis detection among tuberculosis survivors and household contacts by study group (intention-to-treat population) before and after National Tuberculosis Elimination Program policy change that expanded tuberculosis preventive treatment for all household contacts (irrespective of age) ........ 5

Table S3: Median months to tuberculosis diagnosis (intention-to-treat population) ........ 6

Table S4: Form of tuberculosis diagnosed among tuberculosis survivors and household contacts relative to tuberculosis survivors' initial treatment (intention-to-treat population) ........ 7

Table S5: Sociodemographic characteristics of tuberculosis survivors and household contacts (per-protocol population) ........ 8

Table S6: Tuberculosis detection rates and number needed to screen in the per-protocol population actively detected by study visits, by study group ........ 9

Table S7: Sensitivity Analyses: Reclassifying tuberculosis-related deaths among tuberculosis survivors as recurrences, restricted analysis to microbiologically confirmed tuberculosis events, and analysis among participants who received >1 screening visit ........ 10

Figure S1: Kaplan-Meier plots of survival free of tuberculosis diagnosis among household contacts by group. ........ 11

AI Prompts ........ 12

## TB Aftermath Protocol & Additional Information

**Published protocol**: Cox SR, Kadam A, Atre S, Gupte AN, Sohn H, Gupte N, et al. Correction: Tuberculosis (TB) Aftermath: study protocol for a hybrid type I effectiveness-implementation non-inferiority randomized trial in India comparing two active case finding (ACF) strategies among individuals treated for TB and their household contacts. *Trials*. 2024;25(1):178.

**Rationale for non-inferiority margin**: When designing our trial, there was no prior relevant clinical data to support a non-inferiority margin. Thus, based on our conversations with local policymakers, we selected 1·70 per 100 person-years as a clinically meaningful difference that was likely to influence policy, corresponding to 30% reduction in case detection from the more to the less effective intervention. Assuming an average rate of 4·67 per 100 person-years, a difference of 1·70 would imply a rate of (4·67 + 1·70/2) = 5·52 per 100 person-years in the more effective group and (4·67 - 1·7/2) = 3·82 in the less effective group, i.e. a reduction of 1·70 / 5·52 = 30·8% from the more effective group. Our original protocol (Cox et al., Trials 2022) incorrectly reported a non-inferiority margin that applied only to recurrent cases. We noticed this error prior to unblinding the principal investigators and co-investigators or beginning to analyze the primary outcome. The protocol correction was published in 2024 (Cox et al., *Trials* 2024).

**Protocol deviations**: During a data quality control (QC) check, the TB Aftermath study staff identified two instances in which participants randomized to the home-based screening group received the 6-month screening visit at their local clinic instead of at their homes, without proper documentation. We reported the details of these two events to the Johns Hopkins School of Medicine (IRB00247239) and D.Y. Patil Vidyapeeth (DYPV/EC/508/2020 ) Institutional Review Boards (IRBs). Both IRBs acknowledged the protocol deviation notification.

**Secondary outcome**: Our prespecified secondary outcome was originally the proportion of household contacts (HHC) <6 years, by study group, initiated on tuberculosis preventive treatment (TPT) after ruling out tuberculosis disease. However, very few HHC <6 years old initiated TPT in the post-treatment period and thus, a group-wise analysis was not meaningful. Instead of reporting these data in our primary TB Aftermath manuscript, we described barriers to TPT uptake in another manuscript: Kadam A, Moe AH, et al. TB preventive treatment coverage among children under six years of age in TB-affected households. *IJTLD Open*. 2025 Nov 12;2(11):695-697. doi: 10.5588/ijtldopen.25.0207. PMID: 41246466; PMCID: PMC12617088.

## Similar patterns of household clustering of tuberculosis by group (intention-to-treat population)

Of 1076 households, only 7 experienced more than one tuberculosis event.

Home-based screening group: Of 48 households with a tuberculosis survivor who developed recurrence, 3 (6%) also had a household contact who developed tuberculosis. Of 491, households with a tuberculosis survivor who did <u>not</u> develop recurrence, 11 (2%) had a household contact who developed tuberculosis. Thus, the relative risk of tuberculosis among household contacts living with a tuberculosis survivor who did vs. did not experience recurrence was 2·79. Of 14 households with a household contact who developed tuberculosis, 3 (21%) also had a tuberculosis survivor who developed recurrence. Of 525 households with no cases of tuberculosis among household contacts, 45 (9%) had a tuberculosis survivor who developed recurrence. Thus, the relative risk of tuberculosis among tuberculosis survivors living with a household contact who did vs. did not experience tuberculosis was 2·50.

Phone-based screening group: Of 27 households with a tuberculosis survivor who developed recurrence, 2 (7%) also had a household contact who developed tuberculosis. Of 510, households with a tuberculosis survivor who did <u>not</u> develop recurrence, 12 (2%) had a household contact who developed tuberculosis. Thus, the relative risk of tuberculosis among household contacts living with a tuberculosis survivor who did vs. did not experience recurrence was 3·15. Of 14 households with a household contact who developed tuberculosis, 2 (14%) also had a tuberculosis survivor who developed recurrence. Of 523 households with no cases of tuberculosis among household contacts, 25 (5%) had a tuberculosis survivor who developed recurrence. Thus, the relative risk of tuberculosis among tuberculosis survivors living with a household contact who did vs. did not experience tuberculosis was 2·99.

Overall, of 75 households with a tuberculosis survivor who developed recurrence, 5 (7%) also had a household contact who developed tuberculosis. Of 1001, households with a tuberculosis survivor who did <u>not</u> develop recurrence, 23 (2%) had a household contact who developed tuberculosis. Thus, the relative risk of tuberculosis among household contacts living with a tuberculosis survivor who did vs. did not experience recurrence was 2·90. Of 28 households with a household contact who developed tuberculosis, 5 (18%) also had a tuberculosis survivor who developed recurrence. Of 1048 households with no cases of tuberculosis among household contacts, 70 (7%) had a tuberculosis survivor who developed recurrence. Thus, the relative risk of tuberculosis among tuberculosis survivors living with a household contact who did vs. did not experience tuberculosis was 2·67.

**Table S1: Tuberculosis preventive treatment history among enumerated household contacts (intention-to-treat population)**

| | Under six years of age (n=342) | | | Aged six years and above (n=2967) | | | Overall (n=3309) | | |
|---|---|---|---|---|---|---|---|---|---|
| | **Home-based screening group (n=182)** | **Phone-based screening group (n=160)** | **p-value[a]** | **Home-based screening group (n=1489)** | **Phone-based screening group (n=1478)** | **p-value[a]** | **Home-based screening group (n=1671)** | **Phone-based screening group (n=1638)** | **p-value[a]** |
| **Tuberculosis preventive treatment history data available** | | | | | | | | | |
| **Yes** | 165 (91%) | 140 (88%) | 0·38 | 512 (34%) | 471 (32%) | 0·43 | 677 (41%) | 611 (37%) | 0·29 |
| **No** | 17 (9%) | 20 (12%) | | 977 (66%) | 1007 (68%) | | 994 (59%) | 1026 (62%) | |
| **Tuberculosis preventive treatment history (if available)** | | | | | | | | | |
| **Yes** | 96 (58%) | 74 (53%) | 0·41 | 243 (47%) | 191 (41%) | 0·21 | 339 (50%) | 265 (43%) | 0·16 |
| **No** | 69 (42%) | 66 (47%) | | 269 (53%) | 280 (59%) | | 338 (50%) | 346 (57%) | |

a. P-value calculated based on the cluster-adjusted Rao-Scott chi-square test.

**Table S2: Rate of tuberculosis detection among tuberculosis survivors and household contacts by study group (intention-to-treat population) before and after National Tuberculosis Elimination Program policy change that expanded tuberculosis preventive treatment for all household contacts (irrespective of age)**

| | Number of participants in the home-based screening group | Events per person-years in the home-based screening group | Detection rate per 100 person-years[a] in the home-based screening group | Number of participants in the phone-based screening group | Events per person-years in the phone-based screening group | Detection rate per 100 person-years[a] in the phone-based screening group |
|---|---|---|---|---|---|---|
| **Combined (tuberculosis survivors and household contacts)** | | | | | | |
| Before policy change | 1057 | 38/1212 | 2·47(1·67 to 3·53) | 1061 | 25/1250 | 1·52 (0·92 to 2·37) |
| After policy change | 1153 | 26/1350 | 1·93 (1·26 to 2·82) | 1114 | 16/1318 | 1·21 (0·69 to 1·97) |
| **Household contacts** | | | | | | |
| Before policy change | 797 | 8/932 | 0·75 (0·30 to 1·55) | 801 | 9/952 | 0·84 (0·36 to 1·65) |
| After policy change | 874 | 8/1030 | 0·68 (0·27 to 1·40) | 837 | 5/996 | 0·40 (0·11 to 1·03 ) |
| **Tuberculosis survivors** | | | | | | |
| Before policy change | 260 | 30/280 | 10·72 (7·23 to 15·31) | 260 | 16/297 | 5·38 (3·08 to 8·74) |
| After policy change | 279 | 18/320 | 5·62 (3·33 to 8·88) | 277 | 11/322 | 3·41 (1·70 to 6·11 ) |

a. Assumed Poisson distribution to calculate detection rates and group-specific CIs. For tuberculosis survivors and household contacts (combined) and household contacts (alone), we adjusted for household-level clustering.

**Table S3: Median months to tuberculosis diagnosis (intention-to-treat population)**

| Home-based screening group | | | | Phone-based screening group | | | | p-value[a] | Overall | | | |
|---|---|---|---|---|---|---|---|---|---|---|---|---|
| Number of participants | Events | Person-years | Median months to diagnosis (IQR) | Number of participants | Events | Person-years | Median months to diagnosis (IQR) | | Number of participants | Events | Person-years | Median months to diagnosis (IQR) |
| **Combined (tuberculosis survivors and household contacts)** | | | | | | | | | | | | |
| 2210 | 64 (2%) | 2562 | 5·5 (3·6-11·4) | 2175 | 41 (2%) | 2568 | 5·6 (3·3-9·6) | 0·70 | 4385 | 105 (2%) | 5131 | 5·6 (3·4-10·6) |
| **Household contacts** | | | | | | | | | | | | |
| 1671 | 16 (1%) | 1962 | 5·6 (4·1-9·9) | 1638 | 14 (1%) | 1948 | 8·9 (3·5-10·8) | 0·75 | 3309 | 30 (1%) | 3911 | 6·1 (3·9-10·8) |
| **Tuberculosis survivors** | | | | | | | | | | | | |
| 539 | 48 (9%) | 600 | 5·5 (3·3-11·5) | 537 | 27 (5%) | 620 | 5·4 (3·1-8·1) | 0·50 | 1076 | 75 (7%) | 1220 | 5·4 (3·1-9·6) |

a. P-value calculated based on the cluster-adjusted Wilcoxon rank sum test comparing median months to diagnosis in home-based to phone-based screening group.

Abbreviations: IQR= interquartile range

**Table S4: Form of tuberculosis diagnosed among tuberculosis survivors and household contacts relative to tuberculosis survivors' initial treatment (intention-to-treat population)**

| | | Tuberculosis survivors diagnosed with tuberculosis recurrence (n=75) | | | Household contacts diagnosed with tuberculosis (n=30) | | |
|---|---|---|---|---|---|---|---|
| | | Pulmonary only | Extrapulmonary only | Both | Pulmonary only | Extrapulmonary only | Both |
| **Tuberculosis survivor initial tuberculosis episode** | Pulmonary only | 52 | 5 | 0 | 19 | 6 | 1 |
| | Extrapulmonary only | 4 | 13 | 0 | 2 | 2 | 0 |
| | Both | 0 | 0 | 1 | 0 | 0 | 0 |
| | Total[a] | 56 | 18 | 1 | 21 | 8 | 1 |

a. Among the 105 tuberculosis events detected among both tuberculosis survivors and household contacts, 77 (73%) were pulmonary only, 26 (25%) were extrapulmonary only, and 2 (2%) were both.

**Table S5: Sociodemographic characteristics of tuberculosis survivors and household contacts (per-protocol population)**

| | | Tuberculosis survivors | | | Household contacts | | |
|---|---|---|---|---|---|---|---|
| | | **Home-based screening group (n=364)** | **Phone-based screening group (n=471)** | **All (n=835)** | **Home-based screening group (n=565)** | **Phone-based screening group (n=1416)** | **All (n=1981)** |
| Age (years) | | 37 (27-49) | 35 (27-50) | 36 (27-49) | 24 (10-45) | 27 (14-42) | 26 (13-43) |
| Age groups | | | | | | | |
| | < 6 years | NA | NA | NA | 89 (16%) | 143 (10%) | 232 (12%) |
| | 6-17 years | NA | NA | NA | 129 (23%) | 309 (22%) | 438 (22%) |
| | 18-24 years | 65 (18%) | 90 (19%) | 155 (19%) | 73 (13%) | 191 (13%) | 264 (13%) |
| | 25-49 years | 210 (58%) | 262 (56%) | 472 (57%) | 162 (29%) | 532 (38%) | 694 (35%) |
| | ≥50 years | 89 (24%) | 119 (25%) | 208 (25%) | 112 (20%) | 241 (17%) | 353 (18%) |
| Sex | | | | | | | |
| | Female | 171 (47%) | 223 (47%) | 394 (47%) | 329 (58%) | 706 (50%) | 1035 (52%) |
| | Male | 193 (53%) | 248 (53%) | 441 (53%) | 236 (42%) | 710 (50%) | 946 (48%) |
| Setting | | | | | | | |
| | Rural | 118 (32%) | 194 (41%) | 312 (37%) | 142 (25%) | 525 (37%) | 667 (34%) |
| | Urban | 246 (68%) | 277 (59%) | 523 (63%) | 423 (75%) | 891 (63%) | 1314 (66%) |
| Highest education attained[a] | | | | | | | |
| | Illiterate or no formal education | 52 (14%) | 58 (12%) | 110 (13%) | 69/476 (14%) | 180/1273 (14%) | 249/1749 (14%) |
| | Primary school | 132 (36%) | 167 (35%) | 299 (36%) | 219/476 (46%) | 511/1273 (40%) | 730/1749 (42%) |
| | Secondary or high school | 99 (27%) | 146 (31%) | 245 (29%) | 129/476 (27%) | 374/1273 (29%) | 503/1749 (29%) |
| | University or graduate school | 80 (22%) | 100 (21%) | 180 (22%) | 59/476 (12%) | 199/1273 (16%) | 258/1749 (15%) |
| Biomass exposure[b] | | 86 (24%) | 118 (25%) | 204 (24%) | 99 (20%) | 364 (26%) | 463 (24%) |
| Household income (Indian Rupees/month) | | | | | | | |
| | <5000 | 12 (3%) | 27 (6%) | 39 (5%) | 20 (4%) | 42 (3%) | 62 (3%) |
| | 5000-10,000 | 44 (12%) | 78 (17%) | 122 (15%) | 46 (9%) | 192 (14%) | 238 (12%) |
| | ≥10,000 | 306 (84%) | 364 (77%) | 670 (80%) | 435 (87%) | 1176 (83%) | 1611 (84%) |
| Employed (among adults ≥18 years)[c] | | 204 (56%) | 247 (52%) | 451 (54%) | 153/347 (44%) | 529/964 (55%) | 682 (52%) |

a. Children <6 years excluded from denominator (n=89 for home-based screening group and n=143 for phone-based screening group).
b. Includes primary or secondary exposure to unclean cooking fuel such as kerosene or wood. Of 204 tuberculosis survivors with biomass exposure, 151 (74%) were from rural areas (57 [66%] of 86 tuberculosis survivors in the home-based screening group and 94 [80%] of 118 tuberculosis survivors in the phone-based screening group). Of 463 household contacts with biomass exposure, 328 (71%) were from rural areas (54 [55%] of 99 household contacts in the home-based screening group, and 274 [75%] of 364 household contacts in the phone-based screening group).
c. Employed was defined as having at least part-time work (formal or informal contract). Denominator includes 347 household contacts aged ≥18 years from the home-based screening group and 964 from the phone-based screening group.

Data are median (IQR), or n (%). Percentages might not add to 100 due to rounding. Column-wise proportions are provided.
Abbreviations: NA=not applicable
Missingness: <1% missing data for each group: Highest education attained (10/2584 school-aged participants); Household income (11/2816)

**Table S6: Tuberculosis detection rates and number needed to screen in the per-protocol population actively detected by study visits, by study group**

| | Home-based screening group | | | Phone-based screening group | | | p-value[b] |
|---|---|---|---|---|---|---|---|
| | Number of participants | Events | Number needed to screen[a] | Number of participants | Events | Number needed to screen[a] | |
| Combined (tuberculosis survivors and household contacts) | 929 | 19 (2%) | 49 | 1887 | 16 (1%) | 118 | 0·01 |
| Household contacts | 565 | 3 (1%) | 188 | 1416 | 7 (0%) | 202 | 1·00 |
| Tuberculosis survivors | 364 | 16 (4%) | 23 | 471 | 9 (2%) | 52 | 0·04 |
| Tuberculosis survivors treated for extrapulmonary tuberculosis | 132 | 5 (4%) | 26 | 173 | 3 (2%) | 58 | 0·30 |
| Tuberculosis survivors treated for pulmonary tuberculosis | 232 | 11 (5%) | 21 | 298 | 6 (2%) | 50 | 0·09 |

a. Number needed to screen is calculated as the inverse of the proportion of tuberculosis events detected among those screened. Lower number needed to screen indicates higher screening yield.
b. P-value calculated based on the Fisher's exact test to compare the proportion of tuberculosis events detected by group.

**Table S7: Sensitivity Analyses: Reclassifying tuberculosis-related deaths among tuberculosis survivors as recurrences, restricted analysis to microbiologically confirmed tuberculosis events, and analysis among participants who received >1 screening visit.**

| | Number of participants in the home-based screening group | Events per person-years in the home-based screening group | Detection rate per 100 person-years[a] in the home-based screening group | Number of participants in the phone-based screening group | Events per person-years in the phone-based screening group | Detection rate per 100 person-years[a] in the phone-based screening group | Detection rate difference (95% CI)[b] |
|---|---|---|---|---|---|---|---|
| **Intention-to-treat population** | | | | | | | |
| **Reclassified tuberculosis-related deaths[c]** | | | | | | | |
| Combined (tuberculosis survivors and household contacts) | 2210 | 68/2562 | 1·99 (1·48 to 2·62) | 2175 | 44/2568 | 1·25 (0·85 to 1·76) | 0·74 (0·05 to 1·44) |
| Tuberculosis survivors | 539 | 52/600 | 8·66 (6·47 to 11·36) | 537 | 30/620 | 4·84 (3·27 to 6·91) | 3·82 (0·90 to 6·74) |
| **Microbiologically confirmed tuberculosis events only[d]** | | | | | | | |
| Combined (tuberculosis survivors and household contacts) | 2210 | 37/2562 | 1·17 (0·79 to 1·67) | 2175 | 30/2568 | 0·93 (0·60 to 1·39) | 0·22 (-0·35 to 0·78) |
| Household contacts | 1671 | 8/1962 | 0·41 (0·18 to 0·80) | 1638 | 11/1948 | 0·56 (0·28 to 1·01) | -0·16 (-0·59 to 0·28) |
| Tuberculosis survivors | 539 | 29/600 | 4.83 (3·24 to 6·94) | 537 | 19/620 | 3·07 (1·85 to 4·79) | 1·77 (-0·47 to 4·00) |
| **Per-protocol population** | | | | | | | |
| **Microbiologically confirmed tuberculosis events only[d]** | | | | | | | |
| Combined (tuberculosis survivors and household contacts) | 929 | 14/1108 | 1·08 (0·56 to 1·89) | 1887 | 9/2255 | 0·35 (0·15 to 0·70) | 0·73 (0·07 to 1·39) |
| Household contacts | 565 | 3/675 | 0·44 (0·09 to 1·30) | 1416 | 5/1692 | 0·30 (0·10 to 0·69) | 0·15 (-0·42 to 0·71) |
| Tuberculosis survivors | 364 | 11/433 | 2·54 (1·26 to 4·54) | 471 | 4/562 | 0·71 (0·19 to 1·82) | 1·83 (0·17 to 3·48) |
| **Participants who received ≥ 1 visit** | | | | | | | |
| Combined (tuberculosis survivors and household contacts) | 1254 | 20/1466 | 1·16 (0·68 to 1·86) | 2039 | 16/2438 | 0·57 (0·31 to 0·96) | 0·60 (-0·03 to 1·22) |
| Household contacts | 810 | 3/944 | 0·32 (0·10 to 0·93) | 1530 | 7/1831 | 0·38 (0·15 to 0·79) | -0·06 (-0·52 to 0·39) |
| Tuberculosis survivors | 444 | 17/522 | 3·26 (1·90 to 5·21) | 509 | 9/608 | 1·48 (0·68 to 2·81) | 1·77 (-0·05 to 3·60) |
| Tuberculosis survivors treated for extrapulmonary tuberculosis | 162 | 5/190 | 2·63 (0·85 to 6·14) | 191 | 3/228 | 1·32 (0·27 to 3·85) | 1·31 (-1·43 to 4·06) |
| Tuberculosis survivors treated for pulmonary tuberculosis | 282 | 12/332 | 3·61 (1·87 to 6·31) | 318 | 6/380 | 1·58 (0·58 to 3·44) | 2·03 (-0·37 to 4·44) |

a. Assumed Poisson distribution to calculate detection rates and group-specific CIs. For tuberculosis survivors and household contacts (combined) and household contacts (alone), we adjusted for household-level clustering.
b. 95% CI for detection rate difference calculated using normal approximation.
c. Based on verbal autopsies conducted by study staff, reclassified confirmed or probable tuberculosis-related deaths among tuberculosis survivors as recurrences.
d. Microbiologically-confirmed tuberculosis events defined based on India National Tuberculosis Elimination Program records i.e. positive by sputum culture, smear microscopy, and/or rapid molecular testing (i.e., CBNAAT).

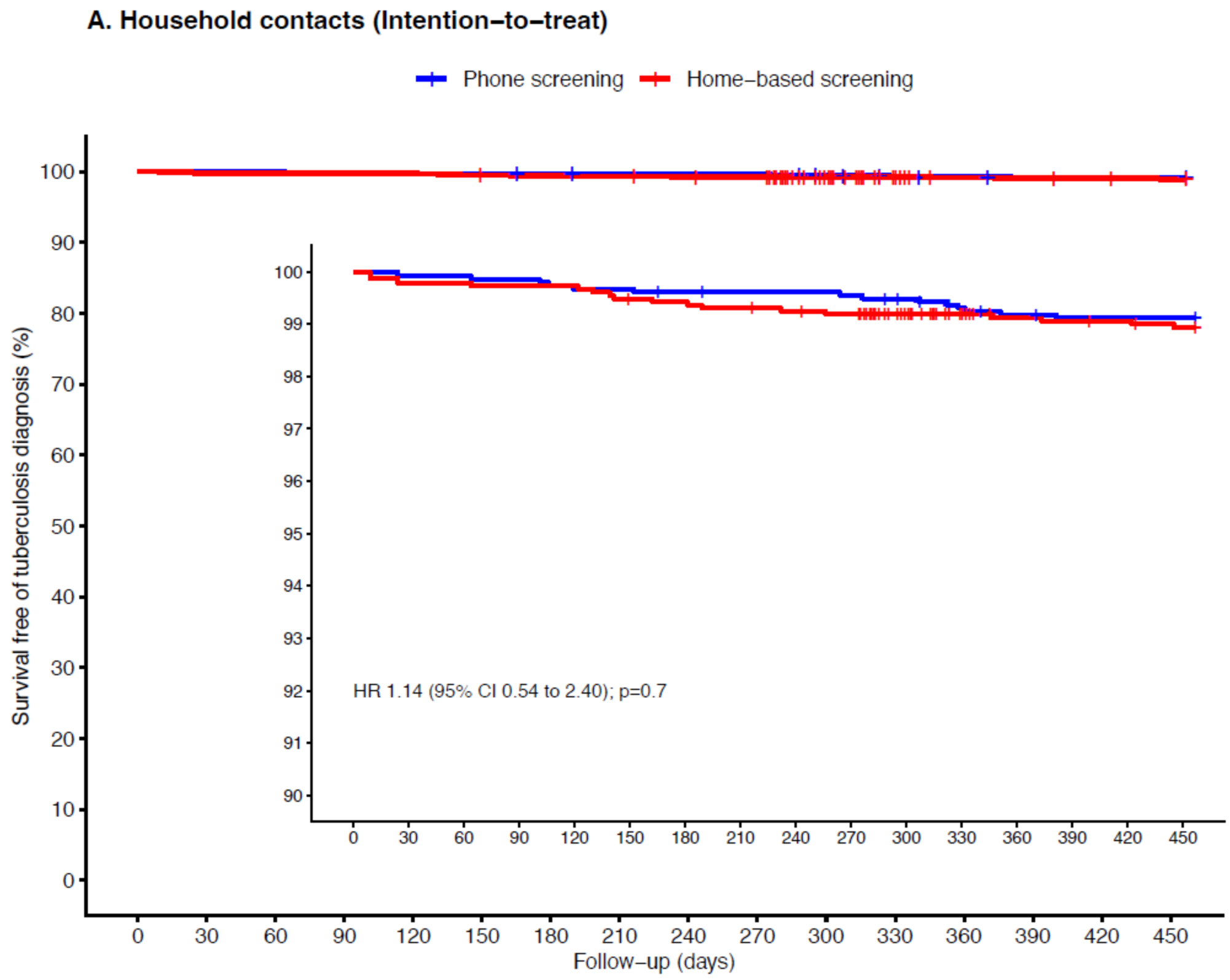


Number at risk

| | | | | | | | | | | | | | | | | |
|---|---|---|---|---|---|---|---|---|---|---|---|---|---|---|---|---|
| Phone screening | 1151 | 1628 | 1628 | 1627 | 1625 | 1625 | 1623 | 1623 | 1623 | 1622 | 1619 | 1616 | 1615 | 1616 | 1616 | 1616 |
| Home–based screening | 798 | 1660 | 1660 | 1660 | 1660 | 1657 | 1656 | 1657 | 1655 | 1653 | 1618 | 1600 | 1592 | 1591 | 1591 | 1588 |

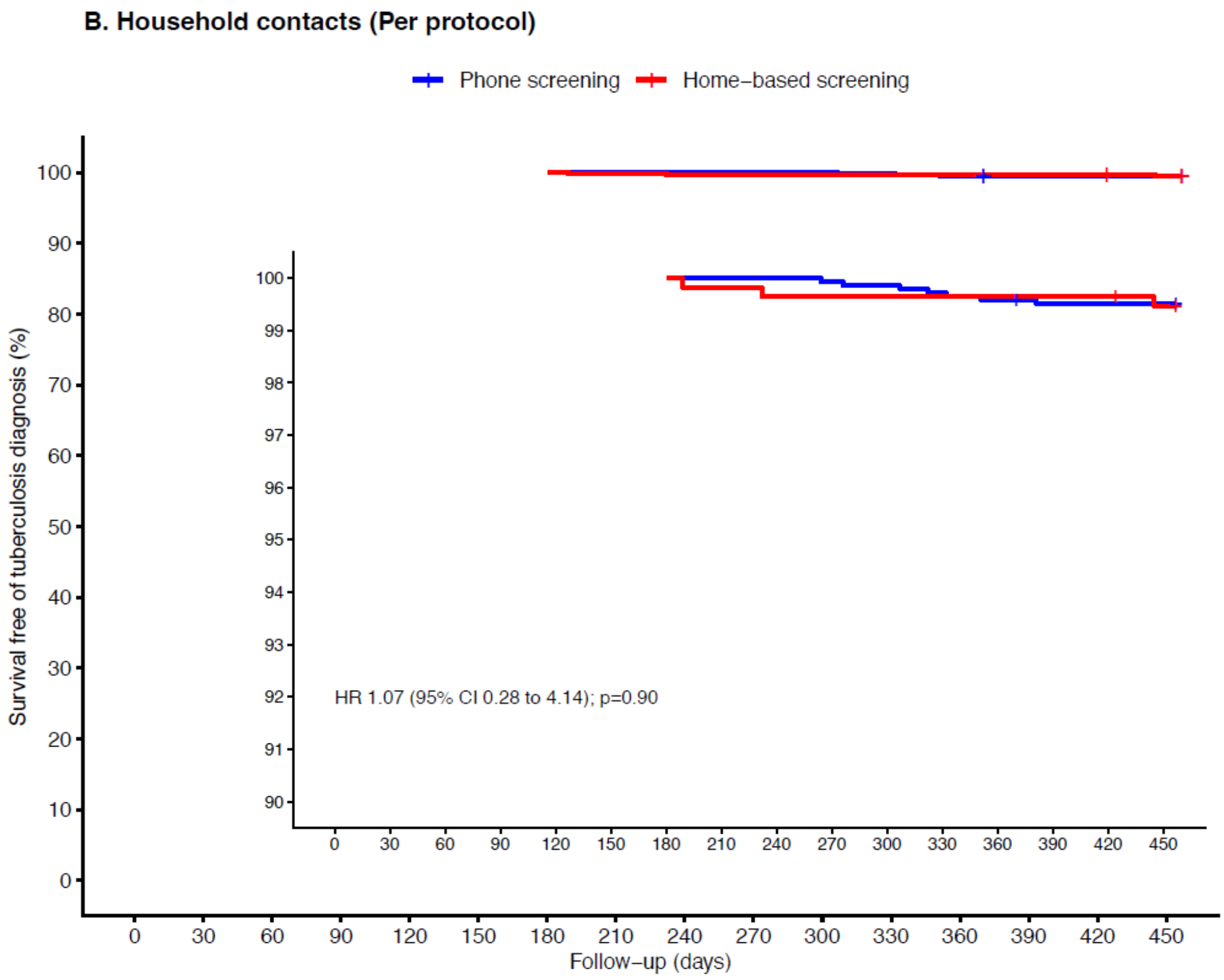


Number at risk

| | | | | | | | | | | | | | | | | |
|---|---|---|---|---|---|---|---|---|---|---|---|---|---|---|---|---|
| Phone screening | 1409 | 1409 | 1409 | 1409 | 1409 | 1409 | 1409 | 1409 | 1409 | 1408 | 1407 | 1407 | 1407 | 1408 | 1408 | 1408 |
| Home–based screening | 564 | 564 | 564 | 564 | 564 | 564 | 564 | 564 | 563 | 563 | 563 | 563 | 563 | 563 | 563 | 561 |

**Figure S1: Kaplan-Meier plots of survival free of tuberculosis diagnosis among household contacts by group.** P-value is calculated based on the robust log-rank test comparing survival free of tuberculosis diagnosis in home-based to phone-based screening group. Panel A: Household contacts only (intention-to-treat population). Panel B: Household contacts only (per-protocol population). Abbreviations: HR=hazards ratio

**AI Prompts**

As declared in the main text, no generative AI tools were used in the writing of this manuscript. We used ChatGPT, OpenAI to assist with troubleshooting issues with R code when generating figures. After using this tool, we reviewed and edited all code and conducted all analysis/interpretation. The authors take full responsibility for the content of the published article.

Below we have specified the types of prompts used:

- "We used ggplot in R with the following code to generate a forest plot: XXXXX. We would like to shift the text on the left farther away from the numbers. Currently they are overlapping. Please modify our code accordingly."
- "We used ggsurvplot in R with the following code to generate our Kaplan Meier curves: XXXXX. We need to add a snapshot of the KM for the 90-100% Y-axis portion of the curve. This snapshot should be displayed within the overall KM to adhere to Lancet guidelines. Please modify our code accordingly."
