# Supplementary material for: Phone-based screening versus home-based screening after tuberculosis in India (TB Aftermath): a multicentre, open-label, randomised, controlled, non-inferiority trial
Source: Lancet Glob Health. Author manuscript; Available in PMC 2026 Oct 20. (PMC13681456; doi:10.1016/j.langlo.2026.103971)
Supplement: Supplementary file 2 [file NIHMS2208782-supplement-2.pdf]

THE LANCET
Global Health

Supplementary appendix 2

This appendix formed part of the original submission and has been peer reviewed. We post it as supplied by the authors.

Supplement to: Cox SR, Kadam A, Valawalkar S, et al. Phone-based screening versus home-based screening after tuberculosis in India (TB Aftermath): a multicentre, open-label, randomised, controlled, non-inferiority trial. *Lancet Glob Health* 2026. https://doi.org/10.1016/j.langlo.2026.103971

Equitable Partnership Declaration

If any questions do not apply to your study, please indicate "N/A" for "not applicable.
For more information on how to complete this form see the Information for Authors document.

Researcher considerations

1. Please detail the involvement that researchers who are based in the country or countries of study had during a) study design; b) clinical study processes, such as processing blood samples, prescribing medication, or patient recruitment; c) data interpretation; and d) manuscript preparation, commenting on all aspects. If they were not involved in any of these aspects, please explain why.

   This should include a thorough description of their leadership roles in the study. Are local researchers named in the author list or the acknowledgements, or are they not mentioned at all (and, if not, why)? Please also describe the involvement of early career researchers based in the location of the study. Some of this information might be repeated from the Contributors section in the manuscript. Note: we adhere to ICMJE authorship criteria for naming authors on a paper.

**a) Study design:** The TB Aftermath co-PI, Dr. Vidya Mave, and Senior Statistician, Dr. Nikhil Gupte, are local researchers based in Pune, India. Both have played critical leadership roles throughout the project. Dr. Mave co-designed the TB Aftermath study alongside Dr. Jonathan Golub, the U.S. co-PI, with inputs from Indian policymakers and co-investigators from India and the U.S. Dr. Gupte performed sample size calculations and wrote the analytical plan during the design phase of the study. Dr. Mave is the co-senior author on our manuscript and Dr. Gupte is a co-author.
**b) Clinical study processes:** Dr. Mave and Dr. Madhu Sudan Barthwal, the lead investigator from D.Y. Patil Vidyapeeth in Pune, India, oversaw all clinical study processes and along with Mr. Abhay Kadam trained study personnel to conduct spirometry, point-of-care HbA1c and COVID-19 antibody testing, and data collection using structured questionnaires and case report forms. Mr. Abhay Kadam is an Indian researcher and the lead study coordinator based in Pune, India for TB Aftermath. Mr. Kadam worked with local study staff to recruit participants, conduct informed consent procedures and follow-up visits, collect data, and coordinate referrals. Ms. Gayatri Lele, Mr. Vipul Dhamale, and Dr. Shahanara Valawalkar are early career researchers from India who were actively involved in data collection, curation, and analysis. Mr. Kadam is the second author on our manuscript and Dr. Barthwal, Ms. Lele, Dr. Valawalkar, and Mr. Dhamale are co-authors. All other local study staff members involved in clinical study processes are named in the acknowledgements and listed in group authorship.
**c) Data interpretation:** The majority of the authors on our manuscript are Indian researchers based in India who contributed to data interpretation throughout the study and during final analyses, conference presentations and manuscript preparation: Abhay Kadam (2nd author), Shahanara Valawalkar (early career), Gayatri Lele (early career), Vipul Dhamale (early career), Nishi Suryavanshi, Arjunlal Kakrani, Nikhil Gupte, Madhu Sudan Barthwal, and Vidya Mave (co-senior author). Co-authors from all collaborating institutions had access to the de-identified datasets. Our primary analysis followed the TB Aftermath study protocol, which was published in Trials in 2022 and approved by our U.S. and Indian IRBs. Preliminary results were discussed on regular conference calls involving team members from the U.S., India, and Korea. Preliminary results were also presented to Indian policymakers from the National TB Elimination Program (NTEP) for feedback in July 2024 (in person) and May 2025 (virtually).

**d) Manuscript preparation:** The manuscript was drafted by Dr. Samyra Cox with mentorship from Dr. Mave (Indian co-PI) and Dr. Golub (U.S. co-PI). All co-authors, including the nine Indian researchers based in India, critically reviewed and edited several drafts of the manuscript and approved the final version. Three of the nine Indian co-authors based in India are early career researchers, as listed above.

2. How was funding used to remunerate and enhance the skills of researchers in the countries of study? And how was funding used to improve research infrastructure at the study sites?

   Potentially effective investments into long-term skills and opportunities within local institutions could include training or mentorship in analytical techniques and manuscript writing, opportunities to lead all or specific aspects of the study, financial remuneration rather than requiring volunteers, and other professional development and educational opportunities.

   Improvements to research infrastructure could include funding extended trial designs (eg, platform trials), establishment of long-term contracts for research staff, building research facilities, and setting up local control of funding allocation.

**Research infrastructure:** The TB Aftermath grant contributed to local research infrastructure improvements through direct funding support to local research institutions based in Pune, India. Specifically, to carry out the TB Aftermath trial, large subawards were extended to Johns Hopkins India (via inter-agency agreement) and Dr. D.Y. Patil Medical College. These subawards contributed to establishing long-term research offices and participant recruitment spaces that can be leveraged for future research studies. TB Aftermath also contributed to local infrastructure improvements by funding the purchase of specialized equipment, such as point-of-care spirometers, that can be utilized by D.Y. Patil to conduct additional research.
**Skills:** Dr. Shahanara Valawalkar is an early-stage clinical researcher based in Pune, India who we were able to recruit for TB Aftermath and who is now on a long-term contract with Johns Hopkins India. Dr. Valawalkar received mentorship and training on conducting trials, managing international research collaborations, collecting verbal autopsy data, and manuscript writing. Ms. Gayatri Lele and Mr. Vipul Dhamale are early-stage statistician who through their involvement in our manuscript, were able to get hands-on experience curating complex trial datasets, carefully handling person-time in survival analyses, and interpreting non-inferiority results. The study staff also received initial and refresher training over the study period for technical aspects of point-of-care spirometry, HbA1c, HSP, GCP, infection control and data management. Data Management staff received training on designing data collection tools in REDCap, basic statistics and their interpretation. Overall, our team of U.S., Indian and Korean co-authors have very complementary skillsets; through our regular meetings and collaborative data interpretation and manuscript writing process, all co-authors had the opportunity to learn from one another and enhance their research skills. Throughout the study period, staff and investigators from India had the opportunity to travel to the Union conference to present preliminary results.

3. How did you safeguard the researchers who implemented the study?

Please describe how you guaranteed safe working conditions for study staff, including provision of appropriate personal protective equipment, protection from violence, and prevention of overworking.

Before the trial was launched, all study staff received training on infection control procedures and appropriate personal protective equipment (PPE) for the clinic-based enrolment visit and for home visits during follow-up. Both participating Indian institutions have implemented well-defined human resources policies. Workloads were monitored to prevent overworking, and staff were employed under formal contracts with clearly defined roles and responsibilities. Additionally, study staff received initial and annual refresher training on workplace sexual and other harassment policies and related HR procedures.

Benefits to the communities and regions of study

4. How does the study address the research and policy priorities of its location?

How were the local priorities determined and then used to inform the research question? Who decided which priorities to take forward? Which elements of the study address those priorities?

The research question evaluating two post-TB screening strategies was developed in collaboration with local policymakers from India's National TB Elimination Program (NTEP). We then presented preliminary results to national-level policymakers from the NTEP in July 2024 during an in-person workshop and solicited feedback on how to interpret the results and next steps for research and policy translation. After incorporating feedback, we then convened a second meeting (virtual) with national-level policymakers from the NTEP in May 2025 to review updated results and gather feedback. In July 2025, we also disseminated preliminary results at the Maharashtra state level in an in-person workshop with state, district and grassroot level TB program staff, local researchers, policymakers, clinicians and study staff (conducted in English and the local language, Marathi).

The first and second authors of our manuscript, Dr. Samyra Cox (U.S.) and Mr. Abhay Kadam (India), co-drafted recommendations for incorporating local stakeholder feedback into the final manuscript. Our co-senior authors, Dr. Vidya Mave (India) and Dr. Jonathan Golub (U.S.), co-led all decisions around how to incorporate local stakeholder feedback into the final manuscript.

5. How will research products be shared in the community of study?

For instance, will you be providing written or oral layperson summaries for non-academic information sharing? Will study data be made available to institutions in the region(s) of study? The Lancet Group *encourages authors to translate the summary (abstract) into relevant languages after paper editing; do you intend to translate your summary?*

We do not currently plan to translate the journal abstract into local languages, as English is widely used for academic and programmatic communication in the study setting.

We will prepare a policy-oriented summary presentation of the final manuscript , similar to our prior NTEP debriefings, where we will share with policymakers from India's National TB Elimination Program (NTEP) at the Maharashtra state level and at the national level. This presentation will be offered in-person and led by local Indian researchers but may be conducted virtually depending on stakeholder preferences. We will also prepare an oral layperson summary of the final manuscript for dissemination to our community advisory board (CAB) for World TB Day. Though we have done this previously, turnover at the NTEP level is common and we want to help influence policy with the new leadership.

6. How were individuals, communities, and environments protected from harm?

a) *How did you ensure that sensitive patient data were handled safely and respectfully? Was there any potential for stigma or discrimination against participants arising from any of the procedures or outcomes of the study?*

All participant information was collected and stored in secure, password-protected databases (REDCap). Personal identifiers were removed or coded for analyses to protect confidentiality. Participant-related confidential paper documents, such as informed consent forms and locator forms, are stored in a double-locked cabinet with an additional biometric lock and are under 24/7 CCTV surveillance in the Data Management Centre at the study site. TB can carry social stigma, so home visits, clinical assessments, and referrals were conducted privately and respectfully at time convenient for participants. Our local counsellors used several strategies to reduce the risk of deepening TB-related stigma including: scheduling home visits at times that were convenient for the participant and their families; visiting homes in plain close without wearing their work badge; conducting study procedures in a private location of the participant's choosing. Additionally, staff were trained using predefined scripts to address issues that might arise because of TB-related stigma

b) *Might any of the tests be experienced as invasive or culturally insensitive?*

Study procedures were conducted with clear explanations, voluntary participation, and informed consent, and did not conflict with local cultural or religious practices. The tests used in the study posed minimal risk (finger prick for HbA1c and Covid-19 antibodies). Importance of these tests, as well as of point-of-care spirometry, was explained during the initial informed consent as well as during each evaluation visits.

c) *How did you determine that work was sensitive to traditions, restrictions, and considerations of all cultural and religious groups in the study population?*

Local study team regularly consult with community advisory boards (CABs) in Pune to ensure that all SOPs for informed consent and follow-up are culturally-appropriate and minimize risk to participants. Additionally, local institutional ethics committee carefully review and

oversee that the research processes are socially and culturally appropriate. Study staff were thoroughly trained in counselling skills and data collection with reference to socio-cultural appropriateness.

d) *Were biowaste and radioactive waste disposed of in accordance with local laws?*

Finger-prick needles, used HbA1c and Covid-19 antibody test kits, antibacterial filters, and consumables such as gloves and masks were appropriately packaged and disposed of at biohazard waste facilities available at TB clinics, rural hospitals, and the medical college. Biohazard waste disposal was an integral part of the standard operating procedures for these tests, and study staff were thoroughly trained in these procedures.

e) *Were any structures built that would have impacted members of the community or the environment (such as handwashing facilities in a public space)? If so, how did you ensure that you had appropriate community buy-in?*

NA

f) *How might the study have impacted existing health-care resources (such as staff workloads, use of equipment that is typically employed elsewhere, or reallocation of public funds)?*

The study was designed to minimize disruption to NTEP services. Routine TB diagnostics were performed by NTEP staff, and study staff coordinated referrals and follow-ups without overburdening clinic workflows.

7. Confirm that local ethics review was sought, and please provide the approval number. If not sought, please explain why.

TB Aftermath was registered with clinicaltrials.gov (NCT04333485) and the Clinical Trials Registry of India (CTRI/2020/05/025059) in 2020. The protocol was approved by the Institutional Review Board (IRB) at Johns Hopkins School of Medicine (IRB00247239) and at our coordinating center in India, D.Y. Patil Vidyapeeth (DYPV/EC/508/2020).

Secondary analyses

8. Have the data analysed in your study been extracted from another source, such as a national survey, rather than being directly collected by the authors of this paper?

Yes / No / N/A

Yes. Primary TB outcome data, including treatment initiation, completion records, bacteriologic results (smear, culture, GeneXpert), and chest X-ray findings, were extracted from the Nikshay program database and paper logs.

If the authors of this paper were not involved in data collection, how were the findings interpreted with sufficient contextual knowledge?
The Lancet Group *believe contextual understanding is crucial for informed data analysis and interpretation*.

NA

---

9. Please provide the title (eg, Dr/Prof, Mr/Mrs/Ms/Mx), name, and email address of an author who can be contacted about this statement.

Name: Dr. Samyra R. Cox
Email: scox26@jh.edu

10. Finally, please provide the title and name of an author from one country of study who has seen and approved this form.

Name: Mr. Abhay Kadam
Email: akadam2@jhmi.edu
